# Self-Assessed Educational Needs of Chinese Pediatric Clinicians Regarding Pediatric Palliative Care: A Cross-Sectional Investigation

**DOI:** 10.3390/children11060730

**Published:** 2024-06-14

**Authors:** Xiaoxia Lu, Yanmei Wang, Jingke Li, Yue-Cune Chang, Niang-Huei Peng

**Affiliations:** 1 School of Nursing and Health, Henan University, Kaifeng 475001, China; livevictory2021@gmail.com (X.L.);; 2 Department of Mathematics, Tamkang University, Tamsui, New Taipei City 25137, Taiwan

**Keywords:** pediatric palliative care, end-of-life care, educational needs

## Abstract

Background: An important first step in enhancing professional palliative care training is to investigate the educational needs of pediatric clinicians in this field. The aims of this research were to analyze the extent of end-of-life care experience of Chinese pediatric clinicians and identify the differing educational needs of physicians and nurses as well as associated impact factors. Methods: A cross-sectional descriptive survey via a structural questionnaire was used in this research. Results: In total, 187 clinicians, comprising 52 physicians and 135 nurses, participated in this study. The topics “pain management”, “symptom management”, and “discussing the prognosis with family members” were the most expressed educational needs among both physicians and nurses. Compared to nurses, physicians placed greater emphasis on the importance of “communication” and “pain and symptom management” (*p* < 0.05). Clinicians with more extensive end-of-life care experience more strongly felt the importance of learning about pain management and communication regarding end-of-life care (*p* < 0.05). Conclusions: Research showed that the education currently provided to pediatric clinicians does not meet their distinctive needs. Future palliative care education must be a continuing multi-level, interdisciplinary program and different education should be provided to physicians and nurses based on their respective needs.

## 1. Introduction

Pediatric palliative care (PPC) is specialized care to prevent and relieve physiological, psychological, social, and spiritual suffering of pediatric patients with life-threatening or life-limiting illnesses and to promote the quality of life of patients and their families. This care extends from the point of diagnosis throughout the ill child’s life, death, and beyond [1]. Although medical advances have significantly improved the survival rate of pediatric patients, each year, in mainland China, many children with life-limiting illnesses and their families continue to experience suffering and death without palliative care services [2]. In mainland China, most pediatric palliative care services are based in hospitals and large cities of the eastern provincial capital; moreover, most existing services mainly serve children with cancer [3,4,5].

The lack of pediatric palliative care education for health professionals has been identified as a significant obstacle to advancing pediatric palliative care in China [2,4,5]. Previous studies have shown that insufficient professional pediatric palliative training for pediatric clinicians could lead to a lack of professional confidence to cooperate with colleagues providing pediatric palliative care for critically ill or terminal patients and their families [6,7]. Additionally, negative emotions may arise when clinicians are faced with the terrible symptoms of patients and suffering of their families, which may affect their job satisfaction, leading to burnout and increased turnover [8].

To remedy this situation, Chinese pediatric clinicians need quality pediatric palliative care training. An important first step in implementing a training program is to identify specific educational needs in this area. The purposes of this study were to (1) analyze the extent of end-of-life care experience of pediatric clinicians and (2) identify the differing educational needs of physicians and nurses, as well as the associated impact factors.

## 2. Methods

A cross-sectional study and a designed questionnaire were used to survey the educational needs of pediatric clinicians regarding palliative care. Based on a survey, most deaths among critically ill children in mainland China occurred in pediatric intensive care units (PICUs) and neonatal intensive care units (NICUs) [2]. Thus, this study investigated the educational needs of clinicians employed in those units. Research samples were recruited via convenience sampling from eight intensive care units at two medical centers and two children’s hospitals in Henan Province in mainland China. The inclusive criteria were physicians and nurses currently employed in a PICU or NICU and who voluntarily participated in this research. Those who did not meet these criteria were excluded. Research data were collected between June 2021 and April 2022.

### 2.1. Research Theory

This study adopted the Education in Palliative and End-of-Life Care Pediatrics Dissemination Model (EPEC Pediatrics Dissemination Model) [9] and the core competencies of pediatric palliative care education proposed by Downing et al. [10] as the theoretical basis for conducting research surveys. The process of education and training for the EPEC Pediatrics Dissemination Model can be divided into (1) needs assessment: understanding the learners’ needs before implementing education; (2) clinical cases: Hook refers to clinical cases introduced prior to education with the goal of triggering learners’ empathy and motivation; (3) the identification of learners’ “attitudes/obstacles/myths/misconceptions” regarding pediatric palliative care practice and the ways to overcome them, e.g., identifying and resolving common misunderstandings in communication among healthcare team, patients, and their families; and (4) knowledge and skills. Additionally, we referred to the core competencies model of pediatric palliative care education to define the educational needs of pediatric healthcare professionals as follows: (1) physical care: pain and symptom management; (2) psychological care: emotional support care for patients and their families; (3) communication: communication issues with care team members, patients, and families; (4) interdisciplinary care team cooperation: collaboration with other health care team members to provide palliative care; (5) ethical issues: clinical and ethical decision making; (6) end-of-life care: identification and care for patients’ terminal symptoms and bereavement support for their families; (7) self-care and self-awareness: education for clinicians regarding self-awareness and self-care abilities when providing palliative care to terminally ill patients and their families.

### 2.2. Research Instrument

The research questionnaire was developed to fit the culture of China with reference to previous studies [11,12]. It comprised three parts: (a) ten items of demographic information and questions related to previous educational experience in pediatric palliative care; (b) seven items on participants’ experience providing various end-of-life care interventions, coded as 1 = never; 2 = rarely; 3 = sometimes; 4 = often; and 5 = always; and (c) ranking thirteen educational topics of pediatric palliative care in order of perceived need.

### 2.3. Reliability and Validity

The content validity of the questionnaire was established by an expert panel comprised of two physicians and three nursing experts. The overall content validity index (CVI) was 0.83, representing the validity of the structured questionnaire. The CVI of experience caring for dying patients was 0.78, and the ranked importance subscale was 0.86. The reliability of the questionnaire was tested using a pilot test on a convenience sample of 20 pediatric clinicians, including 5 physicians and 15 nurses, and the Cronbach alpha for the 10 items on end-of-life care experience was 0.915.

### 2.4. Data Collection Procedure

Participants were voluntarily recruited via convenience sampling. Data were collected via a paper-based questionnaire distributed directly to physicians and nurses in research hospitals during ward meetings and rest times.

### 2.5. Statistical Analysis

Statistical analysis was performed using the statistical package SPSS version 27.0. Fisher’s exact tests, chi-squared tests, and the Mann–Whitney tests were used to analyze research data. A *p* value of less than 0.05 was considered significant in this research.

Based on previous studies, clinicians’ length of experience in end-of-life care impacts their educational needs. As we examined the relationship between educational needs and clinical experience, the median value of participants’ end-of-life care experience was 21. We used the median value (21) as the cut-off point to divide participants into a less experienced group (<21) and a more experienced group (>21). Fisher’s exact tests were used to investigate the impacting effects of clinicians’ previous care experience on their learning needs.

### 2.6. Ethical Considerations

The protocol for this survey was approved by the Human Research and Ethics Committee of the researchers’ university of employment. All participants signed a consent form and were informed that all survey results were anonymous and confidential before completing the questionnaire.

## 3. Results

### 3.1. Demographics of Participants

Out of 196 eligible clinicians, 187 clinicians volunteered to complete the questionnaire, indicating a 95% response rate. This number included 137 (37 physicians and 100 nurses) from two children’s hospitals and 50 (15 physicians and 35 nurses) from two medical centers. Table 1 displays the detailed personal information of participants.

### 3.2. Experience Providing End-of-Life Care for Dying Children

Most participating clinicians reported never having suggested the option of palliative care to the families of dying children, and few had experience discussing with families the issue of providing CPR (cardiopulmonary resuscitation) to their children. Findings show that the participating physicians had more experience in providing end-of-life care than the participating nurses (*p* < 0.05; Table 2).

Additionally, we further compared the experiences of NICU clinicians and PICU clinicians in caring for terminally ill patients (Table 2). The results revealed significantly different levels of experience between NICU and PICU clinicians in the areas of discussing the option of palliative care with parents (Table 2, Question 1, *p* = 0.014) and holding family meetings (Table 2, Question 2, *p* = 0.04), with PICU clinicians being more experienced than NICU clinicians in both areas. However, clinicians in the NICU had more experience recognizing the signs of impending death in terminally ill patients (Table 2, Question 6; *p* = 0.012).

### 3.3. Comparison of Educational Needs

Table 3 compares the educational needs of physicians and nurses. The topics of “pain management”, “management of dying symptoms”, and “discussing with parents regarding patient’s prognosis” were ranked as the top three educational items in which all participants most desired further training. On the other hand, the topics of “providing emotional support to colleagues”, “dealing with negative feelings”, and “dealing with conflicting personal values” were ranked as least important (Table 3).

The participating nurses expressed a stronger desire to learn about “end-of-life symptom management” than the participating physicians (*p* = 0.004; Table 3). On the other hand, physicians more strongly desired to learn about “informing families of the outcomes of current treatment for their dying children” (*p* = 0.034) and “discussing the issue of death with children” (*p* = 0.02) than nurses (Table 3).

Since the literature suggests that NICU and PICU clinicians may display different palliative educational needs based on the characteristics of the patients they treat [11,12], this study also examined the educational needs of 103 NICU clinicians (29 neonatologists plus 74 nurses) and 87 PICU clinicians (23 pediatricians plus 61 nurses). The results revealed that the top-ranked educational needs of both groups were the same.

Clinicians with more extensive experience in end-of-life care tended to feel the importance of learning about pain management more strongly than those with less experience (*p* = 0.018). They also recognized the importance of learning to communicate with colleagues concerning medical treatments for patients more strongly than their less experienced colleagues (Table 4, *p* = 0.003). Moreover, clinicians with more experience showed a greater desire to learn about the issue of communication with patients’ families, such as discussing the ineffectiveness of current treatments (*p* < 0.001) and ethical issues such as whether to perform CPR (*p* = 0.025). Conversely, clinicians with less end-of-life care experience were more concerned with learning to deal with angry family members (*p* = 0.01).

## 4. Discussion

### 4.1. Demographics of Participants

Table 1 displays the detailed personal information of participants. Most reported having received no neonatal or pediatric palliative care education. Compared to physicians, nurses reported receiving less training in palliative care (*p* = 0.013, Table 1). Additionally, few participants agreed that their professional education was sufficient to provide quality end-of-life care, including 26% (*n* = 14) of physicians and 7.4% (*n* = 10) of nurses with a significant difference in agreement (*p* = 0.002).

### 4.2. Experience Providing End-of-Life Care for Dying Children

Since palliative care for children has not yet been widely promoted in clinical practice in mainland China, most clinicians in this research have had little experience in this area. The results (Table 2) may imply that inadequate understanding of pediatric palliative care, inadequate support from colleagues, and lack of clarity about the child’s terminal moment may influence clinicians’ misperceptions of palliative care and lead to strong emotional reactions because most clinicians, patients, and families of dying children may be unprepared to face the death of a child [4,6,13,14,15,16].

### 4.3. Comparison of Educational Needs

According to the results in Table 3, most participants, both physicians and nurses, identified “pain management”, “symptom management”, and “discussing the prognosis with family members” as their three greatest educational needs. These results were likely consistent with previous studies and suggest that participating clinicians value pain and symptom management in end-of-life care and strongly desire more training in these areas [11,17,18].

The participating physicians recognized the importance of the topic “discussing with families the physical distress caused by ineffective treatment” more strongly than nurses (Table 3, *p* = 0.034) or those with less experience did (Topic 5, *p* < 0.001; Table 4). As we know, helping families understand the ineffectiveness of current treatments is an important step in choosing palliative care for critically ill children [19,20]. The literature also revealed that, due to inadequate communication and fear of medical disputes, even if clinicians recognize that ineffective treatment prolongs physical and mental suffering in critically ill children, most tend to repress their own feelings and values, leaving critical medical decisions to the families of patients in hopes of minimizing medical conflicts and disputes [4,16,17,18,19]. This could explain why research participants, particularly physicians and those with more experience in end-of-life care, expressed a need for training in communication skills (Table 3). Due to the influence of traditional cultural customs, Chinese people are usually uncomfortable discussing death and tend to avoid the topic of the death of children [14,19,21].

Communication with families of dying children is a common challenge for clinicians performing palliative care. A study by Zhang et al. also revealed that children who were aware of their medical condition may experience less anxiety or depression than those who were unaware [3]. As our results indicated several differing educational needs between nurses and physicians (Table 3), we suggest that clinical educators should be aware of these differences and provide appropriate education for each group.

Additionally, the results revealed that both NICU and PICU clinicians identified the same three topic-ranked educational needs. It is possible that, due to the number of research participants and geographic location, this survey could not perform a detailed analysis of the different educational needs of the two groups. Because neonatology is a subspecialty of pediatrics, some clinical experts consider that is important to develop an integrated approach to care across these two disciplines [22]. Therefore, further studies may be needed to investigate the educational needs across these two disciplines and conduct a new educational approach for them. However, the results revealed different educational needs between less experienced and more experienced clinicians (Table 4). A recent studyalso demonstrated that the lack of advanced training in hospice care for oncology nurses was a barrier to the implementation of palliative care practices [3].

### 4.4. Suggestions for Future Professional Education and Research

Based on our findings, we make the following suggestions for future professional education and further research:

(1) The principles of hospice and palliative care for children must be incorporated into the basic curriculum of medical and nursing schools [10].
(2) Continuing education must be interdisciplinary and multi-level. Additionally, the hospice and palliative care team should include children’s family members as partners since the purpose of pediatric palliative care is to promote the quality of life for both critically ill children and their families. We suggest that palliative care training be open to family members of ill children. Since pediatric healthcare experts in China also recommend providing palliative care for children in community hospitals or hospice care at home, training must be gradually extended to the general public [3,4].
(3) Educational materials should be developed to meet the educational needs of both professionals and the public. Based on this research, there is an urgent need for different levels of professional training tools and teaching skills to fit the educational needs of all groups [9,10].
(4) Further studies may be needed to investigate the educational needs across pediatric subspecialties and construct a new educational approach for each.

### 4.5. Study Limitations and Strengths

A limitation of this study was that the research setting was limited to hospitals in Henan Province, and the results may not be generalized to represent pediatric clinicians in other provinces in mainland China. Additionally, the closed-ended nature of the questionnaire could lead to self-reporting and responder bias. To remedy the above limitations, further research with enlarged samples, different research settings, and different research methods is needed.

Although several limitations exist, this is the first study to survey educational needs among pediatric clinicians in mainland China.

## 5. Conclusions

This is the first study to survey educational needs among pediatric clinicians in mainland China. The research results revealed the importance of pediatric palliative care education and the differences in needs between physicians and nurses. Research findings contribute to facilitating the planning of educational interventions for medical educators and clinicians.

## Figures and Tables

**Table 1:** Demographic data (N = 187; physicians = 52, nurses = 135; number/percentage).

Item	Physicians		Nurses		*p* Value
Age ^b^					0.020 *
20–25	1	1.9%	10	7.4%	
26–30	13	25.0%	44	32.6%	
31–35	12	23.1%	47	34.8%	
36–40	16	30.8%	24	17.8%	
>40	10	19.2%	10	7.4%	
Gender ^a^					<0.001 *
Female	31	59.6%	131	97.0%	
Male	21	40.4%	4	3.0%	
Hospital of Employment ^a^					0.686
Children’s Hospital	37	71.2%	100	74.1%	
Medical Center	15	28.8%	35	25.9%	
Department					0.906 ^a^
NICU	29	55.8%	74	54.8%	
PICU	23	44.2%	61	45.2%	
Experience in Pediatric or Neonatal Profession (years) ^a^					0.136
0–5	14	26.9%	20	14.8%	
6–10	17	32.7%	66	48.9%	
11–15	14	26.9%	30	22.2%	
>15	7	13.5%	19	14.1%	
Professional Degree					<0.001* ^b^
Associate Bachelor	0	0.0%	37	27.4%	
Bachelor	39 ^c^	75.0%	98	72.6%	
Masters ^d^	13	25.0%	0	0.0%	
Personal Religion					0.141 ^b^
Buddhism or Native Religions	10	19.2%	35	25.9%	
Christianity or Catholicism	1	1.9%	1	0.7%	
No Religion	41	78.9%	99	73.4%	
Marital Status					0.350 ^b^
Married	45	86.6%	106	78.5%	
Never Married	6	11.5%	27	20.0%	
Divorced	1	1.9%	2	1.5%	
Received Educational Training in Palliative Care					0.013 * ^b^
None	39	75.0%	119	88.1%	
Adult Palliative Care Issues	4	7.7%	9	6.7%	
Pediatric Palliative Care Issues	3	5.8%	2	1.5%	
Neonatal Palliative Care Issues	6	11.5%	2	1.5%	
Multiple	0	0.0%	3	2.2%	
Do you consider your professional education on caring for dying pediatric patients and their families to be adequate?					0.002 * ^b^
Strongly/Disagree	2	3.8%	4	3.0%	
Unsure	36	69.2%	121	89.6%	
Strongly/Agree	14	26.9%	10	7.4%	

* *p* < 0.05 ^a^ chi-squared test; ^b^ Fisher’s exact test; ^c^: 5 years of study in medical school for physicians; ^d^: additional 3-year study for physicians in medical school.

**Table 2:** Clinical experiences in providing end-of-life care for dying children (N = 187, 103 NICU clinicians and 84 PICU clinicians).

Items	Occupations	Mean (SD)	Z	*p* Value
1. Discussing with parents the option of the palliative approach for terminally ill children.	52 Physicians	2.35 (0.926)	−5.991	<0.001 *
	135 Nurses	1.48 (0.771)		
2. Holding family meetings to discuss a newly diagnosed life-threatening illness in children.	52 Physicians	1.52 (0.754)	−5.828	<0.001 *
	135 Nurses	1.06 (0.267)		
3. Providing spiritual support for families with dying children.	52 Physicians	2.48 (1.16)	−4.076	<0.001 *
	135 Nurses	1.75 (0.920)		
4. Discussing with family whether the dying child should receive CPR.	52 Physicians	3.02 (1.093)	−7.900	<0.001 *
	135 Nurses	1.53 (0.790)		
5. Providing adequate pain management for terminally ill children.	52 Physicians	2.83 (1.18)	−3.895	<0.001 *
	135 Nurses	2.07 (1.179)		
6. Recognizing the signs of impending death in terminally ill children	52 Physicians	3.20 (1.178)	−4.556	<0.001 *
	135 Nurses	2.28 (1.157)		
7. Providing comfort interventions for terminally ill children.	52 Physicians	3.12 (1.182)	−2.168	0.030 *
	135 Nurses	2.7 (1.271)		

Response code: 1 = never; 2 = rarely; 3 = sometimes; 4 = often; and 5 = always. Data analysis by Mann–Whitney U test. * *p* < 0.05; SD: standard deviation.

**Table 3:** Comparison of the rank-order important educational needs among pediatric clinicians by Fisher’s exact test (N = 187; 52 physicians; 135 nurses; number/percentage).

Education Topics	Physicians			Nurses			*p* Value
	Rank 1–4	Rank 5–8	Rank 9–13	Rank 1–4	Rank 5–8	Rank 9–13	
1. Providing pain control measures	39/75.0%	8/15.4%	5/9.6%	110/81.4%	21/15.6%	4/3.0%	0.215
2. Nursing care for other symptoms at death	26/50.0%	14/26.9%	12/23.1%	95/70.3%	31/23.0%	9/6.7%	0.004 *
3. Discussing the prognosis with family members	28/53.8%	16/30.8%	8/15.4%	73/54.1%	43/31.8%	19/14.1%	0.949
4. Discussing with colleagues that the current treatment is increasing the physical pain of the child.	19/36.5%	23/44.3%	10/19.2%	40/29.6%	55/40.8%	40/29.6%	0.341
5. Informing the family that current treatment will increase the physical distress of the dying child.	24/46.2%	24/46.2%	4/7.6%	50/37.0%	53/39.3%	32/23.7%	0.034 *
6. Discussing with family members whether to perform CPR and assisting family members in making ethical decisions.	22/42.3%	22/42.3%	8/15.4%	56/41.4%	48/35.6%	31/23.0%	0.488
7. Discussing the issue of death with children	8/15.4%	28/53.8%	16/30.8%	14/10.4%	49/36.3%	72/53.3%	0.020 *
8. Providing family grief counseling and supportive care.	9/17.3%	20/38.5%	23/44.2%	20/14.8%	59/43.7%	56/41.5%	0.798
9. Dealing with angry family members.	9/17.3%	13/25.0%	30/57.7%	23/17.0%	53/39.3%	59/43.7%	0.155
10. In the face of the death of children, how do medical staff provide emotional support to each other?	7/13.5%	4/7.7%	41/78.8%	17/12.6%	26/19.3%	92/68.1%	0.152
11. Reconciling your feelings in the face of a child’s death	5/9.6%	5/9.6%	42/80.8%	13/9.6%	16/11.9%	106/78.5%	0.956
12. Dealing with conflicting personal values in the face of family demands for continued ineffective and abusive medical life support.	5/9.6%	16/30.8%	31/59.6%	11/8.1%	43/31.9%	81/60.0%	0.937
13. Providing terminally ill children and their families with end-of-life care interventions that meet their cultural needs	7/13.5%	15/28.8%	30/57.7%	19/14.1%	42/31.1%	74/54.8%	0.973

Rank 1–4 = most important, Rank 5–8 = moderately important, Rank 9–13 = least important.; * *p* < 0.05.

**Table 4:** The impact of care experience on the ranking of educational topics of pediatric palliative care by Fisher’s exact test (N = 187; less professional experience = 95 participants; more professional experience = 92 participants).

13 Education Topics	Less Experience			More Clinical Experience			*p* Value
	Rank 1–4	Rank 5–8	Rank 9–13	Rank 1–4	Rank 5–8	Rank 9–13	
Topic 1	69/72.6%	18/18.9%	8/8.5%	80/87.0%	11/12.0%	1/1.0%	0.018 *
Topic 2	56/58.9%	29/30.5%	10/10.6%	65/70.6%	16/17.4%	11/12.0%	0.111
Topic 3	53/55.8%	31/32.6%	11/11.6%	48/52.2%	28/30.4%	16/17.4%	0.537
Topic 4	22/23.2%	38/40.0%	35/36.8%	37/40.2%	40/43.5%	15/16.3%	0.003 *
Topic 5	32/33.7%	32/33.7%	31/32.6%	42/45.7%	45/48.9%	5/5.4%	<0.001 *
Topic 6	41/43.1%	28/29.5%	26/27.4%	37/40.2%	42/45.7%	13/14.1%	0.025 *
Topic 7	10/10.5%	43/45.3%	42/44.2%	12/13.0%	34/37.0%	46/50.0%	0.524
Topic 8	20/21.1%	38/40.0%	37/38.9%	9/9.7%	41/44.6%	42/45.7%	0.105
Topic 9	24/25.3%	30/31.6%	41/43.1%	8/8.7%	36/39.1%	48/52.2%	0.010 *
Topic 10	16/16.8%	15/15.8%	64/67.4%	8/8.7%	15/16.3%	69/75.0%	0.255
Topic 11	10/10.5%	14/14.7%	71/74.8%	8/8.7%	7/7.6%	77/83.7%	0.251
Topic 12	10/10.5%	31/32.6%	54/56.9%	6/6.6%	28/30.4%	58/63.0%	0.561
Topic 13	18/18.9%	31/32.6%	46/48.5%	8/8.7%	26/28.3%	58/63.0%	0.061

Rank 1–4 = most important; Rank 5–8 = moderately important; Rank 9–13 = least important. The cut point of clinical experience was on the median (21) of experience in the pediatric or neonatal profession. * *p* < 0.05.

## Data Availability

The data that support the findings of this study are available upon request from the corresponding author. The data are not publicly available due to ethical restrictions.
